# ‘Injections-While-You-Dance’: Press Advertisement and Poster Promotion of the Polio Vaccine to British Publics, 1956–1962

**DOI:** 10.1080/14780038.2019.1586061

**Published:** 2019-03-29

**Authors:** Hannah J. Elizabeth, Gareth Millward, Alex Mold

**Affiliations:** a Centre for History in Public Health, London School of Hygiene and Tropical Medicine, London, UK; b Centre for the History of Medicine, University of Warwick, Coventry, UK

**Keywords:** Polio myelitis, British vaccination, public health campaigns, history of emotions

## Abstract

This article discusses the production and dissemination of the emotive and informative messages promoting polio vaccination registration in Britain from 1956–1962 through the lens of public health press advertisements and posters. It argues that as the press reported on the problems which beset the vaccine campaign, and the various publics who could register for the polio vaccination multiplied, the campaign’s content changed. Material was adapted to target the presumed emotional and educational needs of newly eligible publics. The article contends that by attending to the emotional content of this campaign, the variety of publics envisioned by the producers may be examined.

‘Polio could strike your child’. ‘Polio could strike you’. ‘Polio can strike anyone – even the fittest’; so declared three newspaper advertisements from the Ministry of Health’s 1959 campaign to increase rates of polio vaccination in Britain [Figure 1].^1^ Provided to areas where vaccine uptake was low, these advertisements represent snapshots of an evolving vaccination campaign which adapted to persuade an ever broader and more fractured public to seek vaccination. Since the first British epidemic in 1947, polio, a viral disease, had affected thousands of children, sometimes causing muscle weakness, paralysis, or even death. Eye-catching and emotive, newspaper advertisements and posters were just one expression of the government’s polio strategy; disseminating carefully constructed narratives about citizenship, motherhood, youth, and health.^2^ While the campaign’s engagement with these ideas was familiar from other efforts to improve public health,^3^ it also relied on, and battled against, the specific narratives which existed around, and were created by, polio.^4^

**Figure 1.**
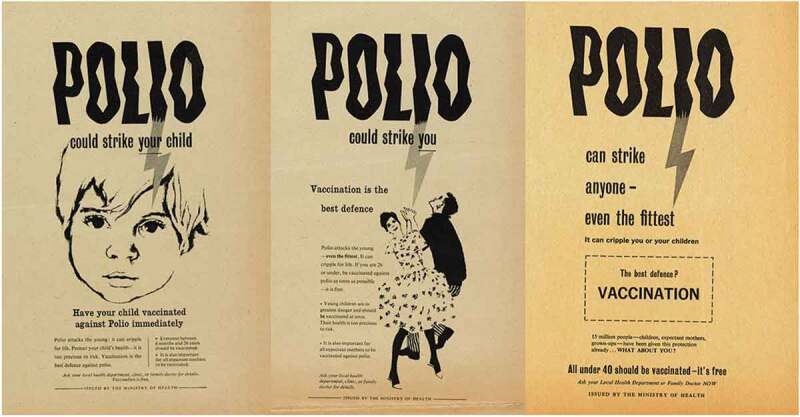
Set of ‘Polio could strike’ posters aimed at ensuring different cohorts were vaccinated, dated left to right 1959, 1959, 1960.

This article explores the government’s polio vaccination campaign through its newspaper advertisements and posters, investigating their cultural, political, and emotional context. It argues that as the press reported on problems which inhibited the vaccine campaign, and the various publics who could register for the polio vaccination multiplied, the posters changed. They evolved to target the assumed emotional and educational needs of each newly eligible public through text and image. The British inactivated poliomyelitis vaccine (IPV) programme began in 1956 and effectively ended in 1962 with the introduction of a new oral vaccine. Initially, IPV – a vaccine using inactivated strains of poliovirus and injected into the patient – was offered only to children. However, the Ministry of Health gradually expanded the eligibility criteria to include young adults and then everyone under the age of 40; an incremental widening of targeted ‘publics’ across the late 1950s and early 1960s. This meant that unlike previous vaccine campaigns which focused on infants, the campaign had to evolve to reach different groups, requiring those producing promotion materials to imagine these publics (and their emotions) in different ways.^5^ This can be seen in the way early poster and newspaper adverts explained IPV’s benefits to the mothers of infants, whereas later propaganda emphasised the financial risks to the family if a father were struck by the disease^6^: the emotionally inflected images and text of the posters were adapted as key target audiences changed. Focusing on the emotional dimensions of the polio vaccination campaign facilitates our exploration of the nature of the relationship between public health policymakers and the public. The perceived need to manage and manipulate the emotions which various ‘publics’ were assumed to possess exposes their constructed nature, but also complicates simplistic understandings of rationality in policy making and practice.

This article discusses the production and dissemination of messages promoting polio vaccination, tracing ideas about both the public and the vaccine back and forth through the archive and into the public sphere via posters and the news print media. This article does not set out to explore the extent to which these materials *worked* in terms of increasing vaccine uptake. The effectiveness of the publicity is difficult to unpick from the impact of the widely reported supply crises and political problems which dogged the vaccine campaign.^7^ Moreover, these have been detailed elsewhere.^8^ Instead, this article performs a close textual analysis of a variety of government issued newspaper advertisements, posters and contemporary newspaper reports to understand the campaign’s emotional context and content. While not exhaustive of all the vaccination educational material produced by the government over the period, they formed the backbone of national (UK-wide) advertisements which were supplemented by local efforts.

We begin with an explanation of our analytic, situating the posters and advertisements as objects methodologically and historically. By making use of a combination of techniques drawn from Bakhtinian literary analysis and the history of emotions, we access some of the emotions behind the textual record of the IPV campaign. This illuminates the government’s evolving assumptions about the various publics they were attempting to persuade to register for the IPV. This approach reveals that the public were a fractured entity, envisioned by the state as groups of rational and irrational emotional communities. As the vaccination programme expanded to include older publics, ideas about IPV, the public and citizenship were overlaid and combined, rather than replaced, allowing a layering of ideas which reached multiple groups through singular texts. Simultaneously, we argue that while it is not our purpose to recover the lived experience of those publics interacting with this campaign, their experiences were, albeit in a limited way, constitutive to the campaign’s trajectory and outcomes. The public were not an inert entity acted on only from above, rather they were a fractured, reactive and unpredictable force which often forced policymakers to respond to their demands for reassurance, information and vaccines.^9^

## Conceptualising publics, texts and emotions

The creation of a ‘text’^10^ for the consumption of the public is an innately political act which transmits both normative and unforeseen ideas about what the public is, should and could be. This perspective, borrowing from Lacan, usefully locates the ends, and audiences, constructed through didactic textual elements; those images and words intended to educate, persuade or dissuade, and which thereby reveal and create the producer’s flawed and ideal imagined audiences.^11^ Bakhtin reminds us texts are artefacts produced not just by their creators, but also by their situated audiences, received within, and changed by, a cultural context in flux. Intertextuality acknowledges this instability and the ‘work-like’ aspects of texts: those dialogical elements which supplement ‘empirical reality by adding to, and subtracting from’ it – the underlying imagination, motives and emotions of audience *and* producer evident in the ideologies and narratives captured by the text and its reception.^12^

Applying this intertextual methodology to public health advertisements and posters is revealing. Posters operate as a particular kind of text. Timmers suggests posters are the ‘product of communication between an active force and a reactive one’; the originator has a ‘message to sell’ which the recipient must be persuaded to ‘buy’.^13^ Indeed, when the poster emerged in the late nineteenth century, its primary subject was advertisements for products available in the growing consumer economy.^14^ Posters were soon deployed for other uses though, and while those delivering public health messages were rare before the First World War, after this period they were increasingly used to communicate messages around issues such as tuberculosis, venereal disease and alcoholism.^15^ Far from neutral texts, Cooter and Stein assert that the public health poster ‘moralizes behavior, guiding the viewer to a clear notion of what is or is not socially acceptable.’^16^ Posters did not simply speak for themselves.^17^ As Gilman reminds us, the vocabulary of posters was complex and contradictory, open to multiple readings that changed over time and place.^18^

While it is difficult to access the experiences of the publics which interacted with posters and advertisements, as Myers points out, pedagogic texts are the product of a ‘two-way traffic between producer and consumer’ and the context in which these are propagated.^19^ Conceiving of texts in this way also provides the tools to excavate the underlying emotions and emotional communities,^20^ both lived and imagined which fostered, produced and were reproduced by the text.^21^ Here, we draw on Rosenwein’s concept of the ‘emotional community’ to guide our analysis. Emotional communities are ‘the same as social communities,’ but as Rosenwein argues, the researcher’s emphasis is upon the ‘systems of feeling’ which govern them – what these ‘communities (and the individuals within them) define and assess as valuable or harmful to them; the evaluations […] about other’s emotions; the nature of the affective bonds between people that they recognize; and the modes of emotional expression that they expect, encourage, tolerate, and deplore.’^22^ An intertextual approach, with its emphasis on the lived experience of both audiences and producers, combined with the attention to emotion Rosenwein encourages, reveals the emotional dialogues expressed within a text, gesturing to the emotional communities (lived and imagined) which form the context for the text’s creation and reception, while emphasising the normative power behind these elements. By understanding the productive work of the makers of polio vaccination posters as taking place within an emotional community, we are able to contextualise the posters’ emotional elements and the work they do as texts. In turn, this close reading can illuminate the authors’ vision of the public as a variety of emotional communities.

These approaches are especially germane to the study of the polio vaccination campaigns because of the volume of comment on the emotions, predominantly fear and anxiety, that surrounded the disease. Indeed, Williams titled his poliomyelitis biography *Paralysed with Fear*.^23^ Yet, despite pervasive acknowledgement of the power of these emotions and their ubiquitous presence within discussions of polio, the British historiography lacks a thorough dealing with its emotional history.^24^ This article begins the work needed to redress this lacuna by exploring which emotions various publics were assumed to be feeling about IPV and which emotions were deployed to persuade them to seek vaccination.

Assumptions about the emotional nature of the public have long played a role in public health policy and practice. As Cantor points out, pre-war medical elites imagined the public as emotionally disturbed, unable to control their behaviour and in danger of being led astray by their emotions.^25^ Until the 1950s, according to Toon, large parts of the ‘cancer establishment’ believed that the public were so afraid of cancer that efforts to educate them about the disease were counterproductive.^26^ Despite these reservations, health education was part of public health practice since at least the nineteenth century, based on the idea that the public could be instructed in rational behaviour. While health education in the inter-war period focused on topics such as personal hygiene, motherhood and the promotion of good citizenship, by the middle of the twentieth century, as infectious disease gave way to chronic disease, campaigns increasingly targeted individual behaviour in order to prevent ill-health.^27^ Vaccination, although still focused on infectious disease rather than new threats to public health like smoking and diet, fitted this preventive ethos.

Efforts to promote vaccination also encapsulated the tension-filled dichotomy between rationality and irrationality often present in health education. The public were imagined as both obstacle and solution to polio – as both rational and emotional – able to act in a way that could prevent polio by registering for the vaccination, or to spread the disease if they failed to vaccinate. This resulted in the construction of different ‘publics’. The public were simultaneously: individuals or groups vulnerable to disease; threats to the stability of the state as potential vectors for the spread of disease; and the mechanism by which, if persuaded to vaccinate, disease might be prevented. Addressing these three publics – potential victim, vector and vaccinator – the government presented the problem of polio as soluble through the actions of obedient citizens. In seeking vaccination, they availed themselves of a new medical technology, guarded the nation against calamity and ensured the possibility of a polio-free future. This narrative of futurity, inherent in preventative medicine, was bolstered throughout the polio vaccine campaign by an emphasis on the availability and newness of the polio vaccine. This was coupled with highlighting vaccination as a duty of citizenship. For parents, this meant protecting children as future citizens.^28^ For young people who could self-register, vaccination operated as a task marking entry into a future as citizens. In the case of older married men, vaccination offered an ensured continuation of their status as breadwinners through the prevention of the potentially incapacitating effects of polio. Thus, vaccination was associated with modern citizenship, an essential part of a desirable lifestyle for young adults and parents alike. Health officials emphasised the benefits of vaccination to the individual, but the undertones were clear. Parents were morally obliged to protect themselves and their children from now-preventable diseases to reduce the risk of disability and expensive medical care in the future. At the same time, vaccination was presented as a ‘gift’ of modern, technocratic medicine and the social rights of the nascent welfare state – albeit a gift that one ought not to refuse.^29^

## Polio, the polio vaccine and emotion

Polio was a ‘virgin soil’ infection of the modern period and the first epidemic did not hit Britain until 1947.^30^ Anxieties were exacerbated by medical science’s inability to offer much defence against the disease.^31^ Indeed, contrary to contemporary public health science, good sanitation and hygiene appeared to make a country more susceptible, with wealthy suburbs and inner-city slums affected in equal measure.^32^ Although most infected people do not present with any symptoms, in severe cases polio can cause limb weakness, paralysis or death.^33^ Therapies were developed to ameliorate acute symptoms (such as the iron lung to combat paralysis of the diaphragm) and long-term rehabilitation (such as callipers and crutches to combat chronic muscle weakness), but no cure was ever found.^34^ The only long-term solution, therefore, was prophylaxis in the form of a vaccine.

The story of polio in Britain was not limited by its national borders – press coverage followed the disease (and attempts combat it) internationally, focusing particularly on the effects of polio, vaccine research and fund-raising efforts in America, such as the March of Dimes.^35^ In 1955, in front of the world’s media, researchers at the University of Michigan announced that they had successfully produced a vaccine based on a formula developed by Jonas Salk, and within hours the US government licenced its use. The British government, traditionally cautious about introducing new vaccines, began trials through its Medical Research Council (MRC), encouraging the *Daily Mail*’s enthusiastic 1955 front-page declaration: ‘Now Polio Vaccine For All – Britain to Buy it for the Health Service’.^36^ This optimism belied the complex web of emotions which circulated around the idea of a polio vaccine. While fear of polio was pervasive in the 1950s, there were also reasons to fear the vaccine. Soon after IPV became widely available in the USA, Cutter Laboratories released a batch of vaccine containing poliovirus which had not been properly inactivated. The incident, which was widely reported by the British press, infected hundreds and led to the deaths of 10 people, shaking the confidence of governments and publics in vaccine manufacturers, doctors, and the arms of government responsible for vaccine rollout.^37^ As the *Daily Express* surmised in 1955, ‘the [American] government stands accused of negligence, manufactures of profiteering, and doctors of running a black market’, while ‘Health Minister Ian Macleod assured M.P.s that all Salk vaccine […] would be most rigorously tested’ and ‘there will be no delay going ahead.’^38^ Fallout from the Cutter incident affected potential IPV programmes globally.^39^ In Britain, the government decided not to abandon IPV, but to insist that a new British formula should be used which would be less prone to manufacturing mistakes, more potent and safer.^40^

Despite the Cutter Incident, there was great appetite for the vaccine in the UK.^41^ Vaccination had become increasingly important in British public health policy since the 1940s. The new health service had begun to provide (or was in the process of testing and licensing) vaccines against diphtheria, whooping cough and tuberculosis.^42^ When Minister of Health Robin Turton finally announced the introduction of the British IPV programme in 1956, the Ministry organised a large press conference to convey the news.^43^ However, the Cutter Incident had forced the Ministry to abandon American IPV and shift to a British formula mere months before the programme was planned to start, meaning that only two companies had licences to manufacture this new type of IPV, and only one had the capacity to deliver the vaccine at the start of the programme.^44^

## Controversy and cohorts

The intention behind the programme was that all children under the age of 10 and certain NHS staff would receive the vaccine – initially in two doses, later three.^45^ To distribute scarce supplies evenly, parents registered their children with the local Medical Officer of Health (MOH) who would then contact the family when IPV became available. The MRC also hoped to test the effectiveness of the vaccine by using the registration scheme to manage a cohort study.^46^ This meant that parents could not simply present their child at the clinic and have the procedure done as with other immunisations. Even if they did register, the MRC’s selection method meant that there was a good chance their child would not be part of the first wave of those to be vaccinated.

Given this complication, it is perhaps unsurprising that registration rates were lower than expected. Moreover, the freedom afforded to MOsH produced variable results. By summer 1957, vaccination rates ranged from 87% in the best-performing district to 20% in the worst (with a national average of 29). Even as supplies became available and the programme was extended to all children under the age of 15 in September 1957, registration was still low. By autumn 1958, it was clear that registration rates were well below the desired coverage in many counties. A nationally co-ordinated publicity campaign would, it was hoped, improve the situation. However, while Ministry officials agreed that ‘stimulus’ in the form of publicity was necessary, they also noted that a ‘national publicity campaign would probably be a waste of effort and money’. Instead, the government believed targeted campaigns in poorly performing areas would be more effective.^47^ Advertisements were placed in the local newspapers of poorly performing districts, while posters were distributed to waiting rooms and other interested parties as they had been during the diphtheria propaganda campaign.^48^

For parents of young children – particularly mothers – vaccination was already a known entity, and government efforts to increase uptake were nothing new.^49^ A national smallpox vaccination programme existed from 1840, and the subsequent success of the war-time diphtheria immunisation campaign had demonstrated the benefits and power of modern vaccine technology.^50^ National and local authorities were similarly experienced in making contact with parents – usually mothers – through advertising and face-to-face contact via health visitors and interactions at clinics.^51^ Debates about falling diphtheria immunisation and subsequent advertising earlier in the decade had established the importance of vaccination and the role citizens ought to play in the maintenance of their family’s health – and had also targeted mothers of young children. Moreover, even before the dedicated winter vaccination promotion campaign of 1958/59, the British public already knew about IPV. There had been consistent press coverage of the disease, the development of this new technology and issues with supply throughout the 1950s.^52^ Locally, MOsH and their staff worked with regional newspapers, community groups and parents to let them know about the vaccine and the procedure for obtaining it.^53^ Thus, when the vaccination registration drive began, the government did not need to create awareness of the vaccine or instil ‘good citizenship’ in the population from scratch.

This context meant that the vaccine and the accompanying education campaign did not enter the target publics’ consciousness unencumbered; nor was the need for vaccination, as comprehended within the Ministry of Health and target health authorities, simply communicated to newly identified target groups in the form of raw epidemiological data. Within the Ministry, the various incarnations of imagined target publics, constructed from epidemiological data then rendered numerically, geographically and according to date-of-birth, were further subdivided into the registered, unregistered and those requiring a third vaccination. These subdivisions identified groups in need of registration and vaccination with exactitude, but were too complex to communicate directly to the public. In fact, publicity materials which explicitly targeted those born after a certain date were abandoned for more simplistic age ranges when it was realised that ‘people did not connect a date of this kind to their own age’.^54^ This moved Ministry campaigns in line with newspapers which reported the vaccine’s availability to new cohorts using age ranges rather than dates of birth.^55^ When advertising efforts included images, much of the imagery and text was designed to do dual service; targeting the under-10s alongside older cohorts in subscript. Posters often captured attention using the salient image of a vulnerable infant, while written encouragements told parents to also seek vaccination for older children.

The vaccination programme and publicity campaign’s traditional focus on young children needed to be balanced with efforts to reach new target publics as the age range of those to be vaccinated expanded. Although there were further supply issues over the course of 1958, by the autumn the Ministry felt it was able to extend the programme. From September, young adults up to the age of 26 were offered the vaccine.^56^ Initially, the Ministry struggled to get young adults interested. They were, however, epidemiologically important. While children were far more likely to contract polio and to become paralysed, adults with polio were more likely to die from the disease.^57^ For adults without young children, the Ministry planned a new publicity campaign to improve uptake. The remainder of this article discusses these developments, excavating the different ways target cohorts were imagined by the campaigns’ producers, and for those members of the public who were assumed to have agency, persuaded.

## Phase 1. ‘they may have cause to reproach themselves’: the 1958/59 winter polio publicity campaign

The 1958/1959 publicity campaign was a response to the uneven national uptake of the polio vaccine; the perception that a ‘good year’ for polio might lead to low press coverage of polio and consequently parental complacency regarding the need to register their children for vaccination; and also a response to direct requests from local health authorities struggling to raise registration rates.^58^ Production of newspaper adverts and posters evolved quickly, moving from text-heavy designs to more striking graphical depictions of children being struck by lightning bolts. A Ministry of Health civil servant explained the intent behind the written and visual elements of the first iteration of the 1958 publicity campaign, admitting the text-heavy advertisement was a little on ‘the long side’ but it was felt that ‘in view of the time element’ they should not plan:

any attempt to use a visual in the press advertisement (which might otherwise be based on one of the photographs of children being vaccinated by a clinic doctor) and get our effect by a quoted extract from a Statement by the Minister set out under a striking heading, with plenty of white space around it, based on the wording of the Minister’s interview with television.^59^

**Figure 2.**
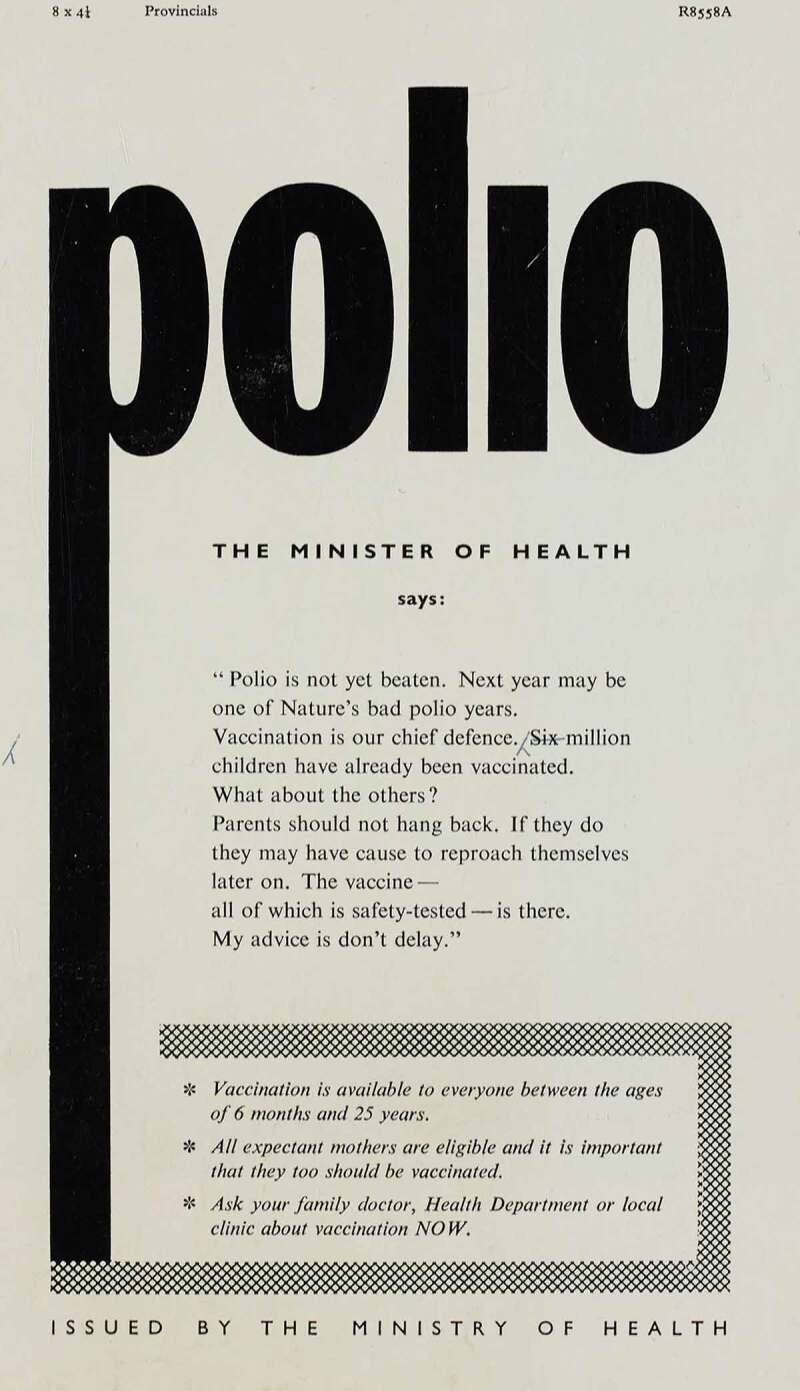
Polio – The Minster of Health says.

The implications behind this letter were that while the more time-consuming addition of a visual element would achieve greater impact, (as would shorter, punchier copy), these shortcomings would not negate the persuasive and informative ‘effect’ of the advertisement if it employed a ‘striking’ title and ‘white space’. Even without the suggested images of children being vaccinated, (or images of children affected by polio, which were typical of the American vaccination campaign), the advertisement’s bold title was eye-catching (see Figure 2).

The opening lines stated that: ‘Polio is not yet beaten. Next year may be one of Nature’s bad polio years. Vaccination is our chief defence.’^60^ These words, chosen from a television interview with the Minister of Health, were more than informative. The advertisement sought to borrow authority from the Minister himself. His words responded to ideas about the polio vaccine already circulating in the public sphere which might reduce vaccine registration, while reiterating existing ideas which might persuade the public to vaccinate. There were two key elements at work here. First, eradicating polio was presented as a battle with ‘Nature’, here depicted as a force given substance through syntax: the possessive apostrophe; the capital ‘N’; and the idea that it held enough agency to be ‘beaten’ imputing a power which rendered those who vaccinated (and those who provided the vaccine) heroic in the face of a fearsome foe. Second, vaccination was presented as a ‘defence’ belonging to a public and government allied against polio and in pursuit of herd immunity through the use of ‘our’, gesturing to the idea of a collective good. Vaccination was part of the social rights bestowed on Britain’s publics, supposedly the hallmarks of the new welfare state era.^61^ But it was also a duty, ‘good’ citizens must vaccinate for the sake of their own (and, by association, the nation’s) children, a concept established during the war with diphtheria immunisation.^62^ This heroic narrative of a manageable present and future threat established, the text then moved to its second main tactic, persuasion through shame and blame:

Nearly seven million children have already been vaccinated.

What about the others?

Parents should not hang back.

If they do, they may have cause to reproach themselves later on.

The vaccine – all of which is safety tested – is there. My advice is don’t delay.

No longer was Nature alone culpable for polio’s scourge. Now parents who ‘hang back’ or ‘delay’ registration and vaccination of their children ‘may have cause to reproach themselves’. The pairing of risk with blame here performs a normative function – encouraging the avoidance of blame through the avoidance of risk. Simultaneously, the risk was rendered manageable and personal, shifting responsibility for polio infection from Nature or the health service, to parents. This transference of responsibility for the unvaccinated was significant given the government’s litany of failures regarding supplies. The statement ‘The vaccine – all of which is safety tested – is there’ dismissed circulating fears around supply issues and the relative safety and efficaciousness of the British and American vaccines.

The public imagined by public health officials and constructed through this advertisement was one requiring instruction and reassurance, but also one open to persuasion. It was a public of adult guardians assumed to have shared emotions and knowledge of polio, aware of the new vaccine, willing to be moved by ideas of children’s vulnerability, conscious of their responsibilities towards their family’s health, but requiring encouragement to take the final step towards vaccination. Children as a public were constructed here not as recipients of the text, but as an affective device; victims of parental failure to act, or mere elements in the ‘defence’ against Nature, the product of a successful alliance of parental and State authority. The child who contracted polio, constructed by the future self ‘reproach’ of parents who failed to vaccinate, was thus rendered a symptom of inadequate parenting and so citizenship – rather than the State’s failure to provide protection. Indeed, fear and blame, not the promise of citizenship, were increasingly used to persuade the public to vaccinate.

## Phase 2. ‘It can leave your child crippled for life’: parents, risk and blame

**Figure 3.**
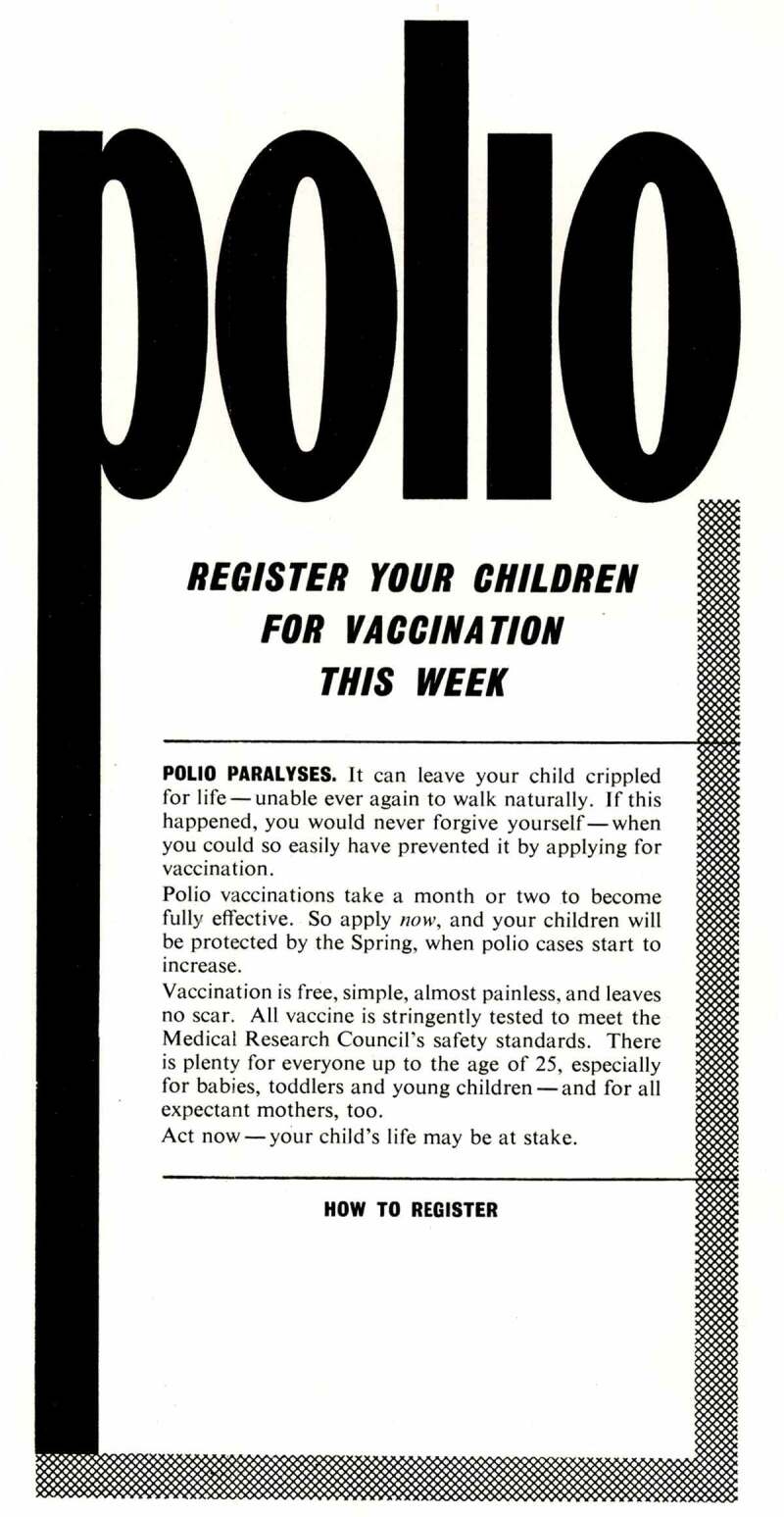
‘You would never forgive yourself’.

At a cursory glance, Figures 2 and 3 appear similar; however, they represent a shift in approach from tentative encouragement (which assumed an obedient public) to a more robust engagement with a public in need of urgent persuasion through fear and shame. Beginning with the subtitle in large bold letters, the advertisement in Figure 3 delivered an imperative message: ‘Register your children for vaccination this week’, giving both an actionable instruction with a deadline and rendering the responsibility of the action personal through the use of ‘your’ rather than the previously favoured collective ‘our’ or plural ‘parents’ deployed in Figure 2.^63^ The desired outcome and responsibility for action established, the poster then proceeded to press parents through fear, shame, reassurance and the provision of information.

The first paragraph of the advertisement (Figure 3) began with the present tense statement ‘Polio Paralyses’, delivered as an immutable fact in bold capitals. This established, it then sketched the grim future which might follow parental failure to seek vaccination registration:

It can leave your child crippled for life – unable ever again to walk naturally. If this happened, you would never forgive yourself – when you could so easily have prevented it by applying for vaccination.

Once again children’s presence within the text was reduced to an emblematic function, a symptom ‘for life’ of a guardian’s failure to ‘easily’ register their child which can ‘never’ be forgiven. The use of em dashes to break the prose forced a pause for meditation upon the idea of the ‘child crippled for life’, the description made more vivid by the layering of additional details – ‘unable ever again to walk naturally’ – which conjured images of mechanical assistance. While the words themselves are emotive, and told the adult audience how to feel, they also relied on the tropes and images of previous representations of polio and its reputation as *infantile* paralysis.

The second and third paragraphs drew on tactics of information and reassurance to encourage guardians to register their children for vaccination, offering timely registration as a rational demonstration of forward planning. While it was explained that immunity was not developed immediately after vaccination – a point likely stressed in response to the upswing in vaccination registration which followed any outbreak of polio despite the vaccine’s inability to provide immediate prophylaxis – reassurance was given; ‘your children will be protected by the Spring, when polio cases start to increase.’ The reassurance continues with

Vaccination is free, simple, almost painless and leaves no scar. All vaccine is stringently tested to meet [MRC] safety standards.

The dismissal of fears around cost, pain and scarring hark back to earlier health procedures, which had to be paid for before the NHS was established or, in the case of smallpox vaccination, free, but rather unpleasant. The reference to ‘stringent testing’ acted as more than a reassurance: it attempted to dismiss lingering fears connected to the Cutter Incident, ongoing confusion around the efficacy and safety of the American versus the British vaccine, and gave an explanation for well publicised delays caused by the destruction of batches of vaccines found to be wanting.^64^ This rationalisation of delay was then followed by ‘There is plenty for everyone up to 25ʹ, a reassurance somewhat diminished by the ‘especially for babies, toddlers and young children – and for all expectant mothers, too’ which followed this statement and belied the continued rationing of the vaccine and growing waiting lists which plagued some boroughs. Indeed, while childless adults under-25 might technically have been able to seek the vaccine through registration, they were not the public addressed or imagined by this poster. The final line of the poster hammers this point home ‘Act now – your child’s life may be at stake’.

## Phase 3. ‘Injections-while-you-dance!’ Vaccination as a marker of agency and healthy citizenship among teenagers, young adults and adult men

Older cohorts, adolescents, and young adults living outside the family unit presented a new challenge to public health authorities who had far less experience advertising vaccination to them. Hitherto, immunisation had been offered to children via parents or specific adults placed at risk through employment. With young adults, the target and the agent making the decision to seek vaccination were one and the same. Yet, they had not typically been the recipients of vaccination and, initially at least, they were reluctant to present themselves, probably because they felt that they were not at risk of polio and did not see the reward of protection against the disease as worth the effort of registering for the vaccine.^65^

**Figure 4.**
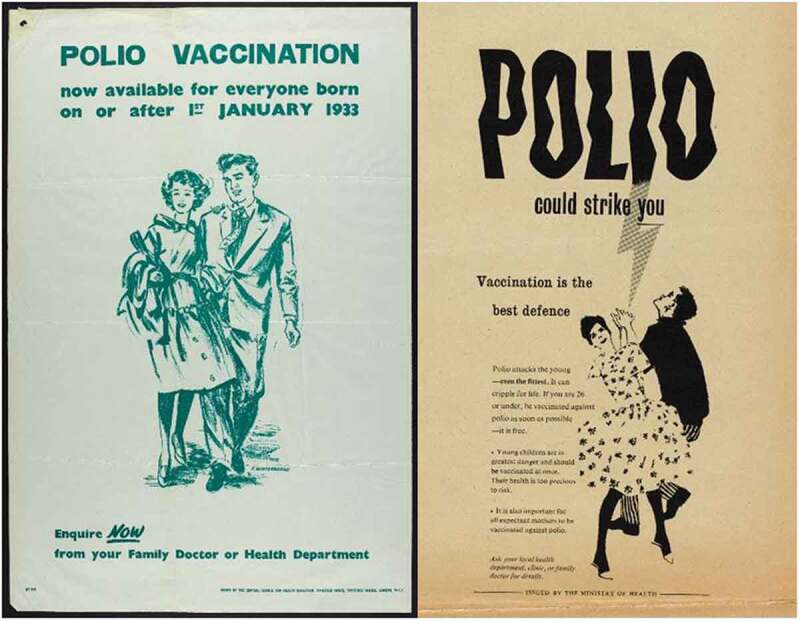
Targeting older cohorts, 1959.

The government responded to the needs of young adults creatively, targeting them where they worked and recreated both literally – by running vaccine drives and placing posters in these spaces – and figuratively through a new set of posters tailored to entice this cohort into registration [see Figure 4]. They began by reusing posters aimed at earlier target audiences, albeit with ‘better colour’.^66^ It was also suggested, however, that the Central Council for Health Education ‘produce another poster specially addressed to the 15–26 age group suitable for display in such places as technical colleges, youth clubs, factories, etc.’^67^ Another idea was to amend a vaccine registration promotion ‘tv filler’ so that it spoke directly to this older cohort.^68^ As with earlier stages, specific efforts were aimed at enticing 15–26s to register for vaccination – but the publicity was designed to target other cohorts too. As Heald explained in a letter regarding posters to local authorities with particularly low uptake: ‘one is primarily directed to the under-16 age group and the other the 16 to 26 age group, but each includes mention of the other and of expectant mothers.’^69^ These efforts multiplied the available discourses surrounding vaccination registration. While ideas of parental responsibility and the threat of paralysis to children still featured – with space given over to telling parents to register children – posters targeted at unattached under 26s were dominated by representations of youthful independence which created an association between vaccination registration and the achievement of rational agency.

By associating vaccination spatially and figuratively with activities which demonstrated independence from the familial unit – work and dancing – posters conveyed the idea that like financial independence and the procurement of a date for a dance, vaccination registration was a marker of maturity and success. The form of citizenship presented here depicted normative values associated with being a modern, young adult in the same way that it had represented ‘good parenting’ when targeting other cohorts. These tactics would eventually come to garner positive media interest, the story no longer entirely about supply issues and failed batches, but the unusual efforts of health authorities to stimulate (and then meet) rising demand amongst the youthful population.^70^

Indeed, there was an increase in uptake of polio vaccination from young people in 1959, but this was the result of an event out of the government’s control. On 4 April 1959, England international footballer Jeff Hall died of polio days after playing for Birmingham City.^71^ That a young, healthy man could die suddenly and apparently at random shocked people into registering for IPV.^72^ The Ministry co-ordinated a message from the Health Minister Derek Walker-Smith at all Football League matches the weekend following Hall’s death, and many clubs took the opportunity to vaccinate their squads in front of national and local media cameras.^73^ The resulting surge in demand caused significant supply problems in certain areas, but in the long run the improved uptake among this cohort was welcomed.

Though Hall’s death undoubtedly led to an increase in vaccination rates, the innovative measures of health authorities across the country should not be overlooked. Injections ‘while you dance’, declared a *Manchester Guardian* article on 8 April 1959, reporting the various efforts of health authorities around the country to reach the under 26s.^74^ The article stated that:

An ‘injections-while-you-dance’ scheme is to be operated in Bristol to encourage immunisation among the under 25s and, in addition to medical teams visiting factories, approaches are to be made to youth and dance halls with a view to give injections to young people. In clubs and halls dance records will be interrupted for propaganda announcements.^75^

An approach that was already part of the ongoing polio vaccination publicity campaign thus became newsworthy in the face of Hall’s death.

## Phase 4 – ‘15 million people.. have been given this protection already. WHAT ABOUT YOU?’ Persuading the 26–40s to vaccinate

It was ironic that, aged 29, Hall would have been too old to be part of the IPV programme in 1959, even if he had sought out vaccination. This, and a number of other factors, meant that the government decided to extend the eligibility criteria to cover all citizens under 40 from January 1960. The reasons for this were threefold. First, the expanded programme had been a pledge in the Conservatives’ 1959 general election manifesto. Despite the supply problems and negative publicity, the programme was considered one of the party’s examples of a commitment to the nation’s health. Second, the Ministry felt capable of delivering IPV now that the majority of under 26s likely to present themselves had done so, and enough children had been vaccinated so that the demand from this group had largely reduced to the annual birth rate. Third, the pharmaceutical company Pfizer had begun to produce IPV from its British plant, improving the supply situation.^76^ However, informed audiences (and interested newspaper readers) knew that the oral polio vaccine being developed by Albert Sabin was likely to be used in the near future.^77^ Sensing that demand for IPV would not last long, Pfizer flooded the market. Caught on the back foot, the Ministry calculated that it would be cheaper for the Treasury to buy the vaccine in bulk for use in the vaccination programme than allow Pfizer to make it available on prescription (where the NHS would have to pay full price for every individual injection offered). This new cohort, then, was created out of specific financial as well as epidemiological circumstances. This cohort was at lower risk of paralysis or death than younger citizens. Still, disability carried significant economic risks which might destabilise the entire family unit; and it was these risks which were addressed by the final stage of the polio advertising campaign.

**Figure 5.**
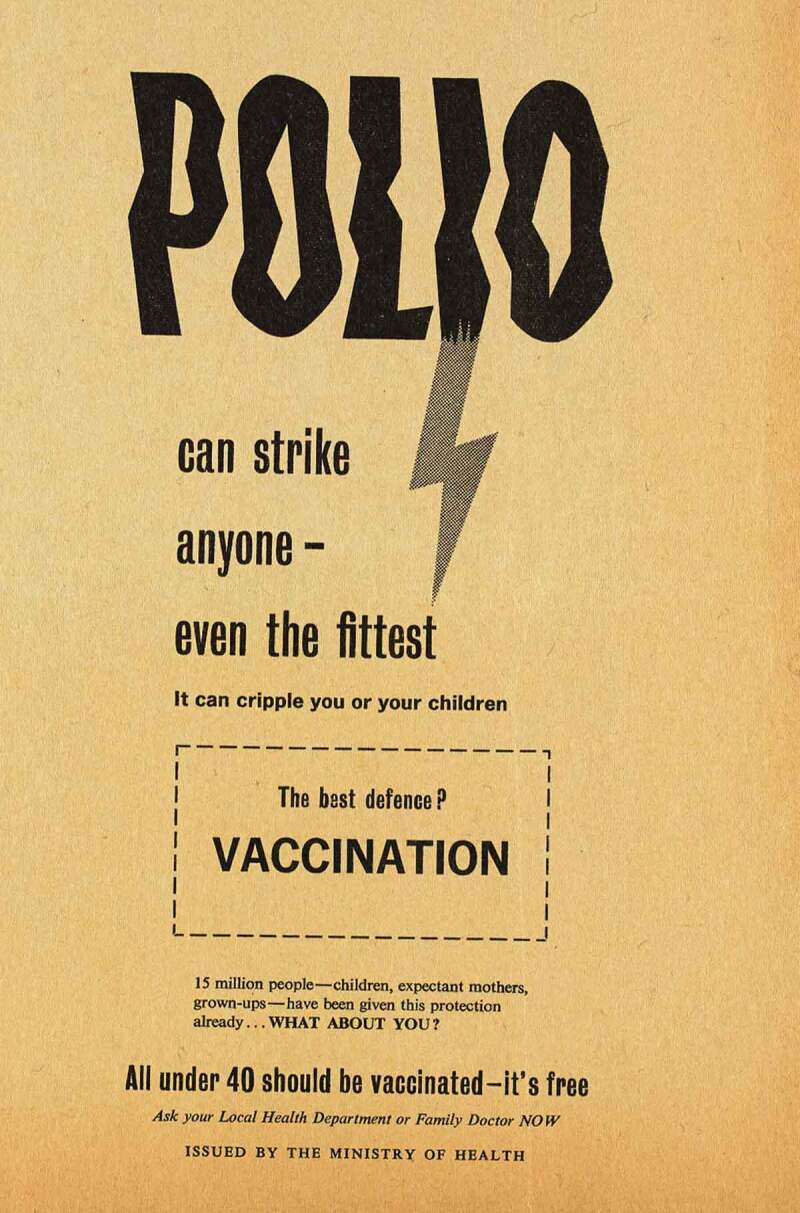
‘15 million people.. have been given this protection already. WHAT ABOUT YOU?’ 1960.

The campaign to encourage the 26–40 cohort to vaccinate themselves intensified across 1961. Posters played upon the ideas which previous iterations had deployed, and they consciously harked back to the success of earlier campaigns. Echoing earlier posters in typeface, tone and through direct quotation, Figure 5 triumphantly announced ‘15 million people – children, expectant mothers, grown-ups – have been given this protection already’ before asking accusingly in bold capitals, ‘what about you?’ Here, the narrative was not so much one of polio’s threat and parental neglect (though these ideas remained important and were generated by words like ‘strike’ and ‘cripple’) but the efficient and expected expansion of the vaccine campaign facilitated by dutiful citizens registering. Citizenship duties for this cohort were not only discussed by the government. Pfizer’s own promotional material shifted the focus away from young-adult socialising and emphasised instead the parental duty, particularly of fathers, to remain healthy bread-winners and administrators of the nuclear household.^78^

**Figure 6.**
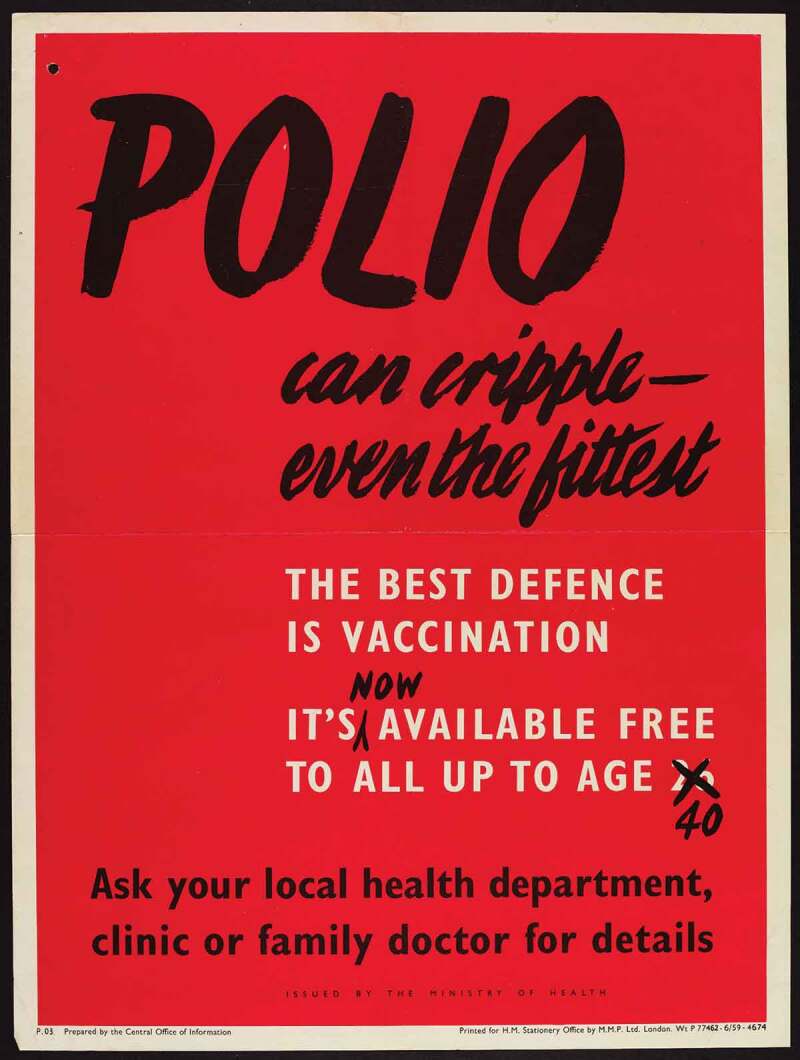
It’s [now] available free to all up to age 26 40.

By overtly signalling previous campaign success alongside the question ‘what about you?’ the advertisement conveyed the idea that expanding vaccination to the 26–40 age group was an inevitable sign of a successfully functioning health system, and that those who failed to avail themselves were reneging on the responsibilities of healthy citizenship which could be easily achieved.^79^ This subtle narrative of inevitable expansion of the campaign and the normalisation of vaccination registration – and so the deviance of failing to vaccinate – was even more explicit in Figure 6.

Appearing to be an annotated reprint of a previously circulated poster, the crossed out number added a hint of humour and signalled government efficiency, redrawing the narrative around vaccination from that of expense, inefficiency and supply issues, to a streamlined natural evolution of a successful health campaign.^80^

While Hall’s death, election promises, and the manoeuvrings of Pfizer might have forced the hand of government to extend the vaccination campaign to the under 40s in a hurry, it was generally met with media approval and ultimately marked a successful end to the attempts of the government to grapple with polio. The change in tone from threat and shame to humour and ubiquity was mirrored in press coverage. Gone were tales of children waiting for vaccinations the government was unable to provide, instead newspapers reported optimistically the expanded cohorts and coming technological innovation of an oral vaccine, declaring ‘Safety on a lump of sugar’^81^ or ‘under 40s will get anti-polio in three lumps’.^82^

## Conclusion

The apparent success of the polio vaccination campaign should not blind us to the problems with its introduction and the anxieties that surrounded the disease throughout the 1950s and beyond. Campaign materials were confident in their presentation of polio vaccination as a way to combat the disease, despite the sometimes chaotic reality of the programme itself and the press’ representation of it as such.^83^ Just as the adverts and posters obscured the disarray that existed on an administrative level, they reveal other, deeper truths. Tracing the emotive content which became a hallmark of this campaign and the interactions of the emotional communities which formed around this disease alerts us to the productive ‘work’ of emotions. Negative emotions such as fear and guilt were played upon within the posters, but positive emotions were also mobilised, such as humour and appeals to young peoples’ burgeoning sense of their own adulthood.

But the work of emotions in this campaign went beyond a simple persuasive tactic intended to increase vaccination uptake. By identifying and building on existing emotional communities, the Ministry of Health were also engaged in creating new groups; the focus on particular target populations transforming emotional communities into a set of overlapping publics. These publics were imagined simultaneously as potential victims, vectors and vaccinators. Unvaccinated children were presented, without agency, as both vectors of disease and victims of parental failure to act as rational citizens. Vaccinating one’s child becoming both an act of good parenting and citizenship. Vaccination was thus a right and a duty: something a parent demanded to protect their child, but also something that they did to ensure collective well-being through the establishment of herd immunity. These narratives around good citizenship highlight the ways in which socio-political rights and responsibilities are emotional, as well as practical and material. Vaccination continues to be an emotive topic, but by assessing the role played by emotions in previous campaigns, we are better placed to understand their function in the present.

## Notes

1. The National Archives (hereafter TNA): MH 55/2212.

2. Gareth Williams, Ray Loadman, *Paralysed with Fear: The Story of Polio* (Basingstoke, 2013).

3. Celia Davies, ‘The Health Visitor as Mother’s Friend’, *Social History of Medicine*, 1(1) (1988), pp. 39–59; Alex Mold, ‘Exhibiting Good Health: Public Health Exhibitions in London, 1948–71’, *Medical History*, 62(1) (2018), pp. 1–26.

4. Charles Rosenberg, ‘What Is Disease?: In memory of Owsei Temkin’, *Bulletin of the History of Medicine*, 77(3) (2003), pp. 491–505, 493–95.

5. Previous vaccination programmes focused on infants or specific birth cohorts. For example, the anti-diphtheria and smallpox programmes were primarily aimed at infants; while the anti-tuberculosis programme was targeted at school leavers. See: Ministry of Health, *Report of the Ministry of Health for the Year Ended 31 December 1956. Part II on the State of the Public Health Being the Annual Report of the Chief Medical Officer for the Year 1956* (Cmnd. 325) (London: HMSO, 1957), pp. 33–35, 96.

6. A similar pattern is noted in the US. See: Paul Offit, *Vaccinated: One Man’s Quest to Defeat the World’s Deadliest Disease* (New York, 2007); David M. Oshinsky, *Polio: An American Story* (Oxford, 2005).

7. Anon, ‘Anti-Polio Rush Hits Supplies’, *Daily Mail*, 18 April 1959; Anon, ‘Rush for Polio Inoculation: Queues in London’, *The Manchester Guardian*, 7 April 1959.

8. Gareth Millward, ‘“A Matter of Commonsense”: The Coventry Poliomyelitis Epidemic 1957 and the British Public’, *Contemporary British History,* 31(3) (2017), pp. 384–406; Ulrike Lindner and Stuart S. Blume, ‘Vaccine Innovation and Adoption: Polio Vaccines in the UK, the Netherlands and West Germany, 1955–1965’, *Medical History* 50(4) (2006), pp. 425–46.

9. Stuart Blume, *Immunization: How Vaccines Became Controversial* (London, 2017).

10. Here the term ‘text’ is used expansively to refer to all artefacts constructed to disseminate ideas, allowing us to analyse words and images in tandem.

11. The methodology draws on a toolkit deployed by Hannah J. Elizabeth in their PhD. See Hannah Kershaw, ‘[Re]inventing Childhood in the Age of AIDS: The Representation of HIV Positive Identities to Children and Adolescents in Britain, 1983–1997’, Unpublished PhD dissertation, University of Manchester, 2016.

12. Dominick Lacapra, ‘Rethinking Intellectual History and Reading Texts’, *History and Theory*, 19(3) (1980), p. 250; Kershaw, ‘[Re]inventing Childhood in the Age of AIDS’, p. 41.

13. Margaret Timmers, ‘Introduction’, in *The Power of the Poster* (London, 1998), pp. 7–25.

14. Paul Rennie, *Modern British Posters: Art, Design & Communication* (London, 2010), 12.

15. William H. Helfand, ‘“Some One Sole Unique Advertisement”: Public Health Posters in the Twentieth Century’, in David Serlin (ed.), *Imagining Illness: Public Health and Visual Culture* (Minneapolis, 2010), pp. 126–42. For examples of posters used in Britain in this period see also Catherine Flood, *British Posters: Advertising, Art & Activism* (London; V&A, 2012) and Hester Vaizey, *Keep Britain Tidy: And Other Posters from the Nanny State* (London, 2014).

16. Roger Cooter, Claudia Stein, ‘Visual Imagery and Epidemics in the Twentieth Century’, in Serlin (ed.), *Imagining Illness*, pp. 169–92.

17. Roger Cooter and Claudia Stein, ‘Coming in to Focus: Posters, Power and Visual Culture in the History of Medicine’, *Medizinhistorisches Journal*, 42 (2007), pp. 180–209.

18. Sander L Gilman, *Health and Illness: Images of Difference* (London: Reaktion Books, 1995).

19. Mitzi Myers, ‘The Erotics of Pedagogy: Historical Intervention, Literary Representation, the “Gift of Education,” and the Agency of Children’, *Children’s Literature*, 23(1) (2009), p. 19.

20. Barbara H. Rosenwein, ‘Worrying about Emotions in History’, *The American Historical Review*, 107(3) (2002), pp. 821–45; Barbara H. Rosenwein, ‘Modernity: A Problematic Category in the History of Emotions’, *History and Theory*, 53(1) (2014), pp. 69–78.

21. By ‘lived and imagined’, we mean those emotions experienced by living publics and those imagined to be constitutive of an imagined public.

22. Rosenwein, ‘Worrying about Emotions in History’, p. 842.

23. Williams, Loadman, *Paralysed with Fear*.

24. For recent histories on polio, see: David M. Oshinsky, *Polio: An American Story* (Oxford, 2005); Paul A. Offit, *The Cutter Incident: How America’s First Polio Vaccine Led to the Growing Vaccine Crisis* (New Haven, 2005); Naomi Rogers, *Polio Wars: Sister Elizabeth Kenny and the Golden Age of American Medicine* (Oxford, 2014). Some work on the history of emotions and polio in America has begun to emerge, however. See: Marc Shell, *Polio and its Aftermath: The Paralysis of Culture* (London, 2005); Daniel J. Wilson, *Polio* (Oxford, 2009).

25. David Cantor, ‘Representing “the Public”: Medicine, Charity and the Public Sphere in Twentieth Century Britain’, in Steve Sturdy (ed), *Medicine, Health and the Public Sphere in Britain: 1600–2000* (London, 2002), pp. 145–68.

26. Elizabeth Toon, ‘“Cancer as the General Population Knows It”: Knowledge, Fear, and Lay Education in 1950s Britain’, *Bulletin of the History of Medicine*, 81(1) (2007), pp. 116–38. On the pre-war period see Ornella Moscucci, ‘The British Fight against Cancer: Publicity and Education, 1900–1948’, *Social History of Medicine*, 23(2) (2009), pp. 356–373.

27. Dorothy Porter, *Health Citizenship: Essays in Social Medicine and Biomedical Politics* (Berkeley, 2011); John Welshman, *Municipal Medicine: Public Health in Twentieth-Century Britain* (Bern, 2000); George Rosen, *A History of Public Health* (New York, 1958); Virginia Berridge, *Marketing Health* (Oxford, 2007).

28. Laura King, ‘Future Citizens: Cultural and Political Conceptions of Children in Britain, 1930s-1950s’, *Twentieth Century British History*, 27(3) (2016), pp. 389–411.

29. Porter, *Health Citizenship*; Frank Huisman and Harry Oosterhout, ‘The Politics of Health and Citizenship: Historical and Contemporary Perspectives’, in Frank Huisman and Harry Oosterhuis (eds), *Heath and Citizenship: Political Cultures of Health in Modern Europe* (London, 2014), pp. 1–40; T. H. Marshall, *Citizenship and Social Class, and Other Essays* (Cambridge, 1950); Richard M. Titmuss, *The Gift Relationship: From Human Blood to Social Policy* (London, 1970).

30. J.N Hays, *Epidemics and Pandemics: Their Impact on Human History* (Santa Barbara, 2005), p. 414.

31. Hays, *Epidemics and Pandemics*.

32. The debate on why this might be so is described in Matthew Smallman-Raynor and Andrew D. Cliff, ‘The Geographical Spread of the 1947 Poliomyelitis Epidemic in England and Wales: Spatial Wave Propagation of an Enigmatic Epidemiological Event’, *Journal of Historical Geography*, 40 (2013), pp. 36–51.

33. Jennifer Hamborsky, Andrew Kroger, and Charles Wolfe (eds), *Epidemiology and Prevention of Vaccine-Preventable Diseases*, 13th ed. (Washington, 2015), pp. 297–310.

34. These efforts were subsidised by public funding and charitable efforts, most notably by the National Fund for Poliomyelitis Research in the UK and the March of Dimes in the US. Rogers, *Polio Wars*; Martyn Hughes, ‘Duncan Guthrie’, *Guardian*, 25 November 1994, p. A21; Wilson, *Polio*.

35. Donald Ludlow, ‘Paralysis Vaccine Tried out: First Tests Passed, But.’, *Daily Express*, 28 March 1953; Anon, ‘America Starts Inocullations of Salk Vaccine: Senator’s Fear of a Black Market’, *The Manchester Guardian*, 14 April 1955; Alistar Cooke, ‘America and Polio:: A Day of Rejoicing’, *The Manchester Guardian*, 16 April 1955; Anon, ‘American Trial Of Salk Vaccine’, *The Times*, 29 April 1955.

36. On British caution on vaccination see: Jane Lewis, ‘The Prevention of Diphtheria in Canada and Britain 1914–1945’, *Journal of Social History* 20(1) (1986), pp. 163–76; Linda Bryder, ‘“We Shall Not Find Salvation in Inoculation”: BCG Vaccination in Scandinavia, Britain and the USA, 1921–1960’, *Social Science & Medicine*, 49(9) (1999), pp. 1157–67; Anon, ‘Now – Polio Vaccine for All: Britain to Buy It for the Health Service’, *Daily Mail*, 26 April 1955.

37. Reuters, ‘New Scare on Salk Vaccine’, *Daily Mail*, 6 June 1955; Anon, ‘Poliomyelitis in US: More Cases Reported After Injections’, *The Times*, 29 April 1955; Reuters, ‘US Firm’s Vaccine Withdrawn: Inquiry into New Cases of Poliomyelitis’, *The Times*, 28 April 1955; Anon, ‘TV Plea on Polio: “Keep Calm”’, *Daily Express*, 30 April 1955.

38. Anon, ‘Polio: “We Accuse” Say Newspapers Over New Vaccine’, *Daily Express*, 3 May 1955.

39. Neal Nathanson, Alexander D. Langmuir, ‘The Cutter Incident Poliomyelitis Following Formaldehyde-Inactivated Poliovirus Vaccination in the United States During the Spring of 1955 II. Relationship of Poliomyelitis to Cutter Vaccine’, *American Journal of Epidemiology*, 78(1) (1963), pp. 29–60; Offit, *The Cutter Incident*; Lindner and Blume, ‘Vaccine Innovation and Adoption’; Alison Day, ‘“An American Tragedy”. The Cutter Incident and Its Implications for the Salk Polio Vaccine in New Zealand 1955–1960’, *Health & History: Journal of the Australian & New Zealand Society for the History of Medicine* 11(2) (2009), pp. 42–61; Per Axelsson, ‘The Cutter Incident and the Development of a Swedish Polio Vaccine, 1952–1957’, *Dynamis*, 32(2) (2012), pp. 311–28; Stuart Blume, *Immunization: How Vaccines Became Controversial* (London, 2017).

40. Gareth Millward, ‘‘A Matter of Commonsense’.

41. See for example National Fund for Poliomyelitis Research ‘People Against Polio’, *The Manchester Guardian*, 18 July 1955, p. 1.

42. Although public health was separate from the National Health Service itself, Section 26 of the National Health Service Act 1946 allowed the Minister of Health to use central funds for the provision of vaccination, administered by local Medical Officers of Health. Smallpox vaccination continued from the nineteenth century, though it was no longer compulsory. See also: Lewis, ‘The Prevention of Diphtheria’; Medical Research Council, ‘Vaccination against Whooping-Cough: Relation between Protection in Children and Results of Laboratory Tests’, *British Medical Journal*, 2(4990) (1956), pp. 454–62; Medical Research Council, ‘B.C.G. and Vole Bacillus Vaccines in the Prevention of Tuberculosis in Adolescents’, *British Medical Journal*, 1(4964) (1956), pp. 413–27.

43. TNA: MH 55/2461, Press Office, Ministry of Health, Polio Vaccine, January 1957; ‘British poliomyelitis vaccine in use this summer’, *The Times*, 20 January 1956, p. 8.

44. Millward, ‘“A Matter of Commonsense”’.

45. Ministry of Health, *Report of the Ministry of Health for the Year 1958. Part II on the State of the Public Health Being the Annual Report of the Chief Medical Officer* (Cmnd. 871) (London, 1959).

46. *Cmnd. 325*.

47. TNA: MH 55/2464, Poliomyelitis Vaccine. Note of a Meeting, 18 September 1958; MH 55/2212, Poliomyelitis Vaccination Estimated Acceptance Rate as at 31 August 1958.

48. Ibid; Distribution invoices and orders are available in TNA: MH 55/2212, e.g. Service Advertising Co. Ltd. invoice to Central Office of Information, 4 November 1958.

49. Angela Davis, Modern Motherhood: Women and Family in England, c. 1945–2000 (Manchester, 2012).

50. Nadja Durbach, *Bodily Matters: The Anti-Vaccination Movement in England, 1853–1907* (Durham, 2005).

51. *Cmnd. 325*, 77–8.

52. See for example the scathing discussion of supply and registration issues from John Starr in the *Daily Mail* where the Minister of Health Robert Turton is accused of misrepresenting Britain’s polio preparedness and a narrative of fear, chronic supply issues, unstable vaccines and queues of vulnerable children are conjured. John Starr, ‘Did Britain Bang the Polio Drum Too Soon?’, *Daily Mail*, 4 April 1957.

53. *Cmnd. 325*, 77–8.

54. TNA: MH 55/2212, ‘Unsigned Memorandum, Poliomyelitis Vaccination Publicity’, 30 January 1959.

55. Ibid.

56. TNA: MH 55/2464, Poliomyelitis Vaccine. Note of a Meeting. 18 September 1958; W.J. Ashcroft [Ministry of Health] to Miss Boys [Treasury], Polio Vaccination. 1 October 1958.

57. Ministry of Health, *Report of the Ministry of Health for the Year Ended 31 December 1955. Part II on the State of the Public Health* (Cmnd. 16) (London, 1956); *Cmnd. 325*; Ministry of Health, *Report of the Ministry of Health for the year 1957. Part II on the State of the Public Health being the Annual Report of the Chief Medical Officer* (Cmnd. 559) (London, 1958).

58. TNA: MH 55/2464, W. N. Judd to Mr. Prideaux, ‘Press Publicity’, 18 September 1958; ibid., W. J. Ashcroft to Miss Boys, ‘Polio Vaccination’, 1 October 1958.

59. TNA: MH 55/2212, Mr. Heald to Mr. Prideaux, ‘Polio Press Advertisement’, 15 October 1958.

60. TNA: MH 55/2212.

61. In particular, see: Marshall, *Citizenship and Social Class, and Other Essays*; Titmuss, *The Gift Relationship: From Human Blood to Social Policy*.

62. Lewis, ‘The Prevention of Diphtheria’.

63. MH 55/2212.

64. Anon, ‘Polio Vaccine Batch Is Condemned, Destroyed’, *Daily Mail*, 29 January 1957; Anon, ‘Vaccine Issue Held Up: Poliomyelitis Scrutiny – Colour Change in Some Samples, Further Tests’, *The Times*, 28 February 1957.

65. Although there was no formal survey done to support this assertion, this was the general attitude taken by the Ministry. The surge in demand caused by the death of footballer Jeff Hall (see below) confirmed this prejudice in the minds of health authorities.

66. Polio Vaccination: Publicity, 13 January 1959.

67. Ibid.

68. Ibid.

69. TNA: MH 55/2212, S.A. Heald to various local authorities, Anti-Poliomyelitis Vaccination Publicity to Raise Acceptance Rate 23 October 1959.

70. ‘Dance Halls Join in Anti-Polio Drive’, *The Manchester Guardian*, 14 April 1959.

71. Anon, ‘Polio vaccine for sportsmen urged’, *The Times*, 6 April 1959, p. 5.

72. Hugh McLeave, ‘Polio Jab Spurt after Hall’s Death’, *Daily Mail*, 4 June 1959.

73. British Movietone, ‘Polio Innoculations’, 13 April 1959. http://www.aparchive.com/metadata/youtube/4bdf81b34ff4492ea60082787a97e807, accessed 27 November 2017; Dennis Holmes, ‘Polio warning goes out to soccer clubs’, *Daily Mail*, 25 March 1959.

74. ‘Injections “While You Dance”’, *The Manchester Guardian*, 8 April 1959.

75. Ibid.

76. Anon, ‘Increasing Supply of Polio Vaccine’, *The Times*, 13 March 1959.

77. Albert B. Sabin, ‘Properties and Behavior of Orally Administered Attenuated Poliovirus Vaccine’, *Journal of the American Medical Association*, 164(11) (1957), pp. 1216–23; Albert B. Sabin, ‘Status of Field Trials with an Orally Administered, Live Attenuated Poliovirus Vaccine’, *Journal of the American Medical Association*, 171(7) (1959), pp. 863–68; Reuters, ‘New Anti-Polio Vaccine: Claims for Dr Sabin’s Work’, *The Manchester Guardian*, October 1956; Anon, ‘Sabin Trials in Manchester: Oral Polio Vaccine’, *The Manchester Guardian*, 17 February 1960.

78. See the collection of Pfizer promotional materials in TNA: MH 55/2212.

79. TNA: MH 55/2212.

80. TNA: MH 55/2212.

81. Hugh McLeave, ‘Polio Shots for UNDER-40’s’, *Daily Mail*, 1 February 1962.

82. Hugh McLeave, ‘UNDER-40’s Will Get Anti-Polio in Three Lumps’, *Daily Mail*, 25 October 1961; Anon, ‘Bad Planning Caused Polio Vaccine Chaos’.

